# Prevention of neural tube defects by the fortification of flour with folic acid: a population-based retrospective study in Brazil

**DOI:** 10.2471/BLT.14.151365

**Published:** 2015-10-27

**Authors:** Leonor Maria Pacheco Santos, Roberto Carlos Reyes Lecca, Juan Jose Cortez-Escalante, Mauro Niskier Sanchez, Humberto Gabriel Rodrigues

**Affiliations:** a Departamento de Saúde Coletiva, Universidade de Brasília, Campus Universitário Darcy Ribeiro, 70910-900, Brasília, DF, Brazil.; b Secretaria de Vigilância em Saúde, Ministério da Saúde, Brasília, Brazil.; c Organização Pan-Americana da Saúde, Brasília, Brazil.; d Faculdades Integradas Pitágoras de Montes Claros, Montes Claros, Brazil.

## Abstract

**Objective:**

To determine if the fortification of wheat and maize flours with iron and folic acid – which became mandatory in Brazil from June 2004 – is effective in the prevention of neural tube defects.

**Methods:**

Using data from national information systems on births in central, south-eastern and southern Brazil, we determined the prevalence of neural tube defects among live births and stillbirths in a pre-fortification period – i.e. 2001–2004 – and in a post-fortification period – i.e. 2005–2014. We distinguished between anencephaly, encephalocele, meningocele, myelomeningocele and other forms of spina bifida.

**Findings:**

There were 8554 neural tube defects for 17 925 729 live births notified between 2001 and 2014. For the same period, 2673 neural tube defects were reported for 194 858 stillbirths. The overall prevalence of neural tube defects fell from 0.79 per 1000 pre-fortification to 0.55 per 1000 post-fortification (prevalence ratio, PR: 1.43; 95% confidence interval, CI: 1.38–1.50). For stillbirths, prevalence fell from 17.74 per 1000 stillbirths pre-fortification to 11.70 per 1000 stillbirths post-fortification. The corresponding values among live births were 0.57 and 0.44, respectively.

**Conclusion:**

The introduction of the mandatory fortification of flour with iron and folic acid in Brazil was followed by a significant reduction in the prevalence of neural tube defects in our study area.

## Résumé

**Objectif:**

Déterminer si l'enrichissement en fer et en acide folique des farines de blé et de maïs (devenu obligatoire au Brésil depuis juin 2004) est efficace dans la prévention des anomalies du tube neural.

**Méthodes:**

À partir des données des systèmes nationaux d'information sur les naissances enregistrées dans les régions du Centre, du Sud et du Sud-est du Brésil, nous avons déterminé la prévalence des anomalies du tube neural parmi les naissances vivantes et les mortinaissances, sur une période préalable à l'obligation d'enrichissement (de 2001 à 2004) et sur une période postérieure à cette obligation (de 2005 à 2014). Nous avons fait la distinction entre les cas d'anencéphalie, d'encéphalocèle, de méningocèle, de myéloméningocèle et des autres formes de spina bifida.

**Résultats:**

Entre 2001 et 2014, 8 554 anomalies du tube neural ont été recensées pour 17 925 729 naissances vivantes. Sur cette même période, 2 673 anomalies du tube neural ont été enregistrées pour 194 858 mortinaissances. La prévalence générale des anomalies du tube neural est passée de 0,79 pour 1 000 avant l'obligation d'enrichissement à 0,55 pour 1 000 postérieurement à cette obligation (rapport de prévalence, RP: 1,43; intervalle de confiance de 95%: 1,38-1,50). Concernant les mortinaissances, la prévalence est passée de 17,74 pour 1 000 mortinaissances avant l'obligation d'enrichissement à 11,70 pour 1 000 postérieurement à cette obligation. Concernant les naissances vivantes, les valeurs correspondantes ont été de 0,57 et 0,44 respectivement.

**Conclusion:**

Au Brésil, l'introduction de l'obligation d'enrichissement en fer et en acide folique des farines a été suivie d'une baisse significative de la prévalence des anomalies du tube neural dans la zone que nous avons étudiée.

## Resumen

**Objetivo:**

Determinar si el enriquecimiento de la harina de trigo y maíz con hierro y ácido fólico (obligatorio en Brasil a partir de junio del año 2004) es efectivo en la prevención de los defectos del tubo neural.

**Métodos:**

Haciendo uso de los datos de los sistemas de información nacionales respecto a nacimientos en el centro, sudeste y sur del Brasil, se determinó la prevalencia de los defectos del tubo neural entre los nacidos vivos y los mortinatos en el periodo previo al enriquecimiento, es decir, de 2001 a 2004, y en el periodo posterior al enriquecimiento, es decir, de 2005 a 2014. Se distinguió entre la anencefalia, el encefalocele, la meningocele, el mielomeningocele y otras formas de espina bífida.

**Resultados:**

Hubo 8.554 defectos del tubo neural entre los 17.925.729 nacidos vivos notificados entre 2001 y 2014. Durante el mismo periodo, se registraron 2.673 defectos del tubo neural entre los 194.858 mortinatos. La prevalencia general de los defectos del tubo neural cayó del 0,79 por cada 1.000 nacimientos antes del enriquecimiento al 0,55 por cada 1.000 nacimientos después del enriquecimiento (índice de prevalencia: 1,43; intervalo de confianza (IC) del 95%: 1,38–1,50). En el caso de los mortinatos, la prevalencia cayó del 17,74 por cada 1.000 mortinatos antes del enriquecimiento al 11,70 por cada 1000 mortinatos después del enriquecimiento. Los valores correspondientes entre los nacidos vivos fueron de 0,57 y 0,44 respectivamente.

**Conclusión:**

La introducción del enriquecimiento obligatorio de la harina con hierro y ácido fólico en Brasil tuvo como resultado una reducción significativa de la prevalencia de los defectos del tubo neural en el área de estudio.

## ملخص

**الغرض:**

تحديد ما إذا كانت عملية تعزيز القمح ودقيق الذرة بإضافة الحديد وحمض الفوليك – والتي أصبحت أمرًا إلزاميًا في البرازيل ابتداءً من يونيو/حزيران من عام 2004 – هي عملية فعّالة في الوقاية من عيوب الأنبوب العصبي.

**الطريقة:**

باستخدام بيانات مأخوذة من نظم المعلومات الوطنية عن المواليد في وسط وجنوب شرق وجنوب البرازيل، قمنا بتحديد معدلات انتشار عيوب الأنبوب العصبي بين المواليد على قيد الحياة ووفيات الرضّع، في فترة ما قبل الإثراء – أي من عام 2001 إلى عام 2004 – وفي فترة ما بعد الإثراء – أي من عام 2005 وحتى عام 2014. ولقد قمنا بالتمييز بين انعدام الدماغ، والقيلة الدماغية، والقيلة السحائية، والقيلة النخاعية السحائية، وغيرها من أشكال السنسنة المشقوقة.

**النتائج:**

لقد كانت هناك 8554 حالة مصابة بعيوب الأنبوب العصبي من بين 17,925,729 من المواليد على قيد الحياة الذين أبلغ عنهم في الفترة بين عامي 2001 و 2014. وبالنسبة لنفس الفترة، تم الإبلاغ عن 2,673 حالة مصابة بعيوب الأنبوب العصبي من بين 194,858 من حالات وفيات الرضّع. انخفض المعدل العام لانتشار عيوب الأنبوب العصبي من 0.79 لكل 1000 حالة قبل الإثراء إلى 0.55 لكل 1000 حالة بعد الإثراء (نسبة الانتشار: 1.43؛ وبنسبة أرجحية مقدارها 95%: 1.38–1.50). أما بالنسبة لوفيات الرضّع المبكرة، فقد انخفض معدل الانتشار من 17.74 لكل 1000 حالة وفيات مبكرة للرضّع قبل الإثراء إلى 11.70 لكل 1000 حالة وفيات مبكرة للرضّع بعد الإثراء. ولقد كانت القيم المقابلة بين المواليد على قيد الحياة 0.57 و 0.44 على التوالي.

**الاستنتاج:**

إن تقديم عملية الإثراء الإلزامية للدقيق باستخدام الحديد وحمض الفوليك في البرازيل أعقبه انخفاض كبير في معدلات انتشار عيوب الأنبوب العصبي في المنطقة التي خضعت للدراسة الخاصة بنا.

## 摘要

**目的:**

旨在确定含铁和叶酸的强化小麦和玉米面粉（巴西已从 2004 年起强制添加）是否在预防神经管缺陷症方面有效。

**方法:**

通过使用巴西中部、东南部和南部地区的国家生育信息系统数据，我们确定了强化前（即：2001 年至 2004 年间）和强化后（即：2005 年至 2014 年间）神经管缺陷症在活胎和死胎间的患病率。我们已对先天无脑畸形、脑膨出、脊膜膨出、脊髓脊膜膨出和其它形式的脊柱裂做了区分。

**结果:**

在 2001 年至 2014 年间公布的 17 925 729 个活胎中，有 8554 个患有神经管缺陷症；同一时期，194 858 个死胎中，有 2 673 个报告患有神经管缺陷症。神经管缺陷症的整体患病率由千分之** **0.79** **降至千分之** **0.55【患病率比值** **(PR)： 1.43；95%** **置信区间： 1.38-1.50】。死胎患病率从强化前的千分之** **17.74** **降至强化后的千分之** **11.70；活胎对应值分别为** **0.57 和** **0.44。

**结论:**

在我们研究领域内，巴西在面粉中强制添加铁和叶酸的强化举措促使神经管缺陷症患病率的显著降低。

## Резюме

**Цель:**

Определить, способствует ли обогащение пшеничной и кукурузной муки железом и фолиевой кислотой, являющееся обязательным в Бразилии с июня 2004 г., профилактике дефектов нервной трубки.

**Методы:**

С помощью данных о рождаемости, полученных из национальных информационных систем в Центральной, Юго-Восточной и Южной Бразилии, была определена распространенность дефектов нервной трубки среди живорожденных и мертворожденных детей до введения обязательного обогащения, т. е. в период 2001–2004 гг., и после него, т. е. в период 2005–2014 гг. Были выделены анэнцефалия, энцефалоцеле, менингоцеле, миеломенингоцеле и другие формы незаращения дужек позвонков.

**Результаты:**

Было зарегистрировано 8554 дефекта нервной трубки у 17 925 729 живорожденных детей в период с 2001 по 2014 г. В этот же период было зарегистрировано 2673 дефекта у 194 858 мертворожденных детей. Общая распространенность дефектов нервной трубки снизилась с 0,79 на 1000 детей (значение до введения обязательного обогащения) до 0,55 на 1000 детей (значение после введения обязательного обогащения) (коэффициент распространенности, КР: 1,43; 95% доверительный интервал, ДИ: 1,38–1,50). Среди мертворожденных детей распространенность снизилась с 17,74 на 1000 детей (значение до введения обязательного обогащения) до 11,70 на 1000 детей (значение после введения обязательного обогащения). Аналогичные значения среди живорожденных детей составили 0,57 и 0,44 соответственно.

**Вывод:**

После введения обязательного обогащения муки железом и фолиевой кислотой в Бразилии распространенность дефектов нервной трубки в области проведения нашего исследования существенно уменьшилась.

## Introduction

Neural tube defects, which are detected in about 300 000 neonates worldwide each year, are a major cause of neonatal morbidity and mortality.^1^ They are caused by the abnormal closure of the embryonic neural tube between 22 and 28 days after conception. The resulting structural defects, which may occur anywhere along the neuraxis, often lead to the postpartum exposure of neural tissue and this, in turn, may lead to severe impairment in the child’s physical and mental development.^2^^,^^3^

Classically, neural tube defects are divided into two main groups: defects affecting brain structures – such as anencephaly and encephalocele and defects that affect the structures of the spinal cord – such as meningocele, myelomeningocele and other forms of spina bifida.^4^^,^^5^ Anencephaly – also called exencephaly or craniorachischisis – is defined as the complete or partial absence of the brain. This defect, which is caused by a failure of the cephalic neural tube to close,^6^^,^^7^ leads to fetal deaths, stillbirths or neonatal deaths. In encephalocele, the brain and meninges herniate through a skull defect, especially in the occipital region.^1^ Spina bifida is characterized by the failure of fusion of the vertebral arches of the spine. This defect can be covered by skin – when it is known as spina bifida occulta – or be associated with a cystic protrusion. In meningocele, this protrusion contains abnormal meninges and cerebrospinal fluid. In myelomeningocele, the cystic protrusion contains elements of the spinal cord and/or nerves.

Neural tube defects have been associated with both genetic factors – e.g. simple gene mutations and chromosomal abnormalities^7^ – and maternal factors such as folate intake, age, ethnicity, obesity and the use of antiepileptic drugs.^8^ If maternal intake of folic acid can be increased around the time of conception, the risk of the occurrence of neural tube defects may be reduced by 60–70%.^9^^–^^12^ This preventive strategy has been adopted by 78 countries^13^ that have mandated the addition of folic acid to flour. Such folic acid fortification has already been associated with reductions in the prevalence of neural tube defects in Canada,^14^ Chile,^15^ South Africa^16^ and the United States of America (USA).^17^

In 2002, Brazil’s Health Surveillance Agency made the fortification of wheat and maize flour with iron and folic acid mandatory in the country from June 2004 – allowing flour producers more than a year to adapt to the new legislation^18^ All wheat and maize flour sold in Brazil since June 2004 should contain 0.15 mg folic acid per 100 g.^18^

Although most studies on the prevalence of neural tube defects have been focused on live births, the prevalence of such defects among miscarriages and stillbirths may be higher than that among live births. In the United Kingdom of Great Britain and Northern Ireland, for example, such defects were detected in 2.8/1000 live births and 5.3/1000 miscarriages at eight weeks of gestation.^19^ In Northern Ireland and south-east England neural tube defects were 7.1/1000 in live births and 10.8 /1000 in eight-week miscarriages.^20^

In this population-based retrospective study, we aimed to determine if the mandatory addition of folic acid to flour sold in Brazil was associated with a change in the prevalence of neural tube defects in live and stillbirths.

## Methods

### Databases

We analysed data that had been routinely collected, in central, south-eastern and southern Brazil, by the national ministry of health and recorded within either the live birth information system database – i.e. as live births – or the mortality information system database – i.e. as stillbirths.

The live birth database was introduced in 1990 but has only included notification of congenital anomalies since 1999. Between 1999 and 2006, only one of the codes of the *International classification of diseases* *and related health problems*^21^ (ICD) could be entered in this database per birth. Since 2007, however, it has been possible to enter multiple codes to cover all of the congenital anomalies present in a child.

### Study design

We compared the prevalence of neural tube defects among live births and stillbirths in the pre-fortification period – i.e. 2001–2004 – with that recorded in the post-fortification period – i.e. 2005–2014. In our analyses, we assumed that all children born in 2004 would have been conceived before fortification became mandatory in June 2004. We therefore considered the whole year of 2004 to be pre-fortification.

### Defect prevalence

We identified as neural tube defects all anomalies coded Q00, Q01 or Q05 according to the ICD, tenth revision,^21^ indicative of anencephaly, encephalocele and all forms of spina bifida, respectively.

We only analysed data from the states of central, south-eastern and southern Brazil in which the proportion of all births registered in either the live birth database or the mortality database was estimated to exceed 95%.^22^^,^^23^ The eight states we included were the central states of Distrito Federal and Mato Grosso do Sul, the south-eastern states of Espírito Santo, Rio de Janeiro and São Paulo and the southern states of Paraná, Rio Grande do Sul and Santa Catarina.

### Study variables

**Fig. 1:**
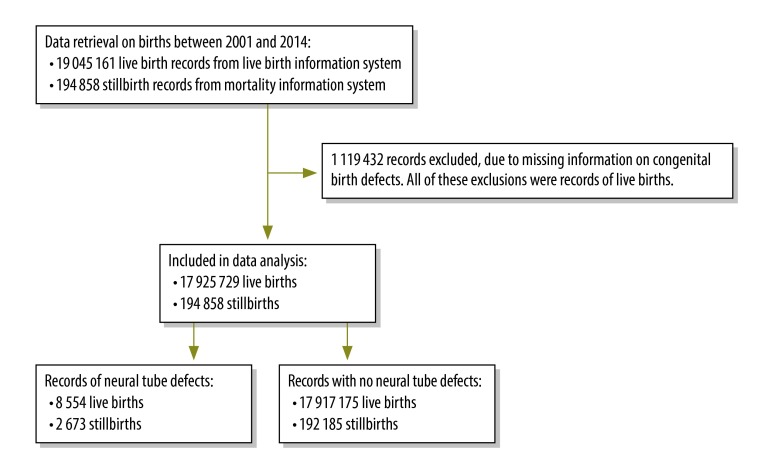
Flowchart for the selection of birth records from the national live birth information and mortality information systems, Brazil, 2001–2014

In our analysis we used flour fortification as the independent variable, the presence of at least one neural tube defect as the dependent variable, and maternal age, sex of the neonate or stillborn child and race of the neonate as the predictors. If no information on outcome of a birth had been recorded – i.e. if it was possible that a neural tube defect had been detected but not recorded – that birth was excluded from our analyses (Fig. 1). Maternal age was categorized into three age groups: younger than 20, 20 to 34 and older than 34 years – representing adolescents, young adults and older mothers, respectively. Neonatal race was recorded in the live birth database as black, brown, indigenous or white. No information on stillbirth race was available from the mortality database. The data available on maternal age and sex of the neonates or stillbirths were incomplete.

### Data analysis

Data were exported and analysed using Epi Info version 7.0 (Centers for Disease Control and Prevention, Atlanta, USA) and Origin 6.0 (Microcal Software, Northampton, USA). The prevalence of any neural tube defect and of defect subtypes coded Q00, Q01 and Q05 were each estimated for the pre-fortification and post-fortification periods. The ratio between each pre-fortification prevalence and the corresponding post-fortification value – and the relevant 95% confidence interval (CI) – was calculated.

### Ethics

The study protocol was approved by the ethics committee of the Universidade Estadual de Montes Claros, Montes Claros, Minas Gerais, Brazil.

## Results

We investigated the records of 19 045 161 live births from the live births database and the records of 194 858 stillbirths from the mortality database. All of the births we investigated occurred between 1 January 2001 and 31 December 2014 and together they represented 45.5% of the total (42 285 756) birth records in Brazil for that period. The 17 925 729 valid cases of live births and 194 858 stillbirths used in our analyses included 8554 and 2673 cases of neural tube defects, respectively (Fig. 1).

Table 1 summarizes the prevalence of neural tube defects recorded among all live births and stillbirths. In the pre-fortification period, the prevalence of such defects among all births – i.e. live births and stillbirths combined – was 0.79 per 1000 births. The corresponding post-fortification value was 30.1% lower, at 0.55 per 1000 births. Among the live births, the prevalence of neural tube defects was 22.8% lower post-fortification than pre-fortification: 0.44 versus 0.57 per 1000 live births. Among stillbirths, the prevalence was 34.0% lower post-fortification than pre-fortification: 11.70 versus 17.74 per 1000 stillbirths.

**Table 1:** Prevalence of neural tube defects before and after the mandatory fortification of flours with folic acid, Brazil, 2001–2014

Type	No. of births investigated	No. of births with NTD	Prevalence		
			NTD per 1000 births	Ratio (95% CI)	Change, %
**Live births**					
Pre-fortification^a^	4 938 343	2 823	0.57	1.29 (1.24–1.35)	−22.8
Post-fortification^b^	12 987 386	5 731	0.44	1	
Total	17 925 729	8 554	–	–	–
**Stillbirths**					
Pre-fortification^a^	65 121	1 155	17.74	1.52 (1.40–1.63)	−34.0
Post-fortification^b^	129 737	1 518	11.70	1	
Total	194 858	2 673	–	–	–
**All births**					
Pre-fortification^a^	5 003 464	3 978	0.79	1.43 (1.38–1.50)	−30.1
Post-fortification^b^	13 117 123	7 249	0.55	1	
Total	18 120 587	11 227	–	–	–

CI: confidence interval; NTD: neural tube defect.

^a^ The pre-fortification study period ran from 1 January 2001 to 31 December 2004 – although fortification became mandatory in June 2014.

^b^ The post-fortification study period, which ran from 1 January 2005 to 31 December 2014, was used as the reference category in the calculation of prevalence ratios.

The prevalence of spina bifida and, particularly, anencephaly were lower post-fortification than pre-fortification among both live births and stillbirths (Table 2). Although we found the prevalence of encephalocele among stillbirths to be higher post-fortification than pre-fortification, this defect was only reported for 39 stillbirths.

**Table 2:** Prevalence of anencephaly, encephalocele and spina bifida before and after mandatory folic acid fortification of flour, Brazil, 2001–2014

Type of birth defect	Pre-fortification^a^			Post-fortification^b^		Prevalence change, %
	No. of cases	Prevalence, cases per 1000 births		No. of cases	Prevalence, cases per 1000 births	
**Live births**						
Anencephaly	1035	0.21		2063	0.16	−24.2
Encephalocele	388	0.08		871	0.07	−14.6
Spina bifida	1400	0.28		2832	0.22	−23.1
**Stillbirths**						
Anencephaly	1056	16.22		1322	10.19	−37.2
Encephalocele	39	0.60		95	0.73	22.3
Spina bifida	80	1.23		123	0.95	−22.8
**All births**						
Anencephaly	2091	0.42		3385	0.26	−38.3
Encephalocele	427	0.09		966	0.07	−13.7
Spina bifida	1480	0.30		2955	0.23	−23.8

^a^ The pre-fortification study period ran from 1 January 2001 to 31 December 2004 – although fortification became mandatory in June 2014. We analysed the data for 4 938 343 live births and 65 121 still births that occurred during this period.

^b^ The post-fortification study period ran from 1 January 2005 to 31 December 2014. We analysed the data for 12 987 386 live births and 129 737 still births that occurred during this period.

The apparent effects of maternal age and sex and race of the child on the prevalence of neural tube defects are summarized in Table 3. In the pre-fortification study period, compared with mothers aged 20–34 years, adolescents were 34% more likely to give birth to a still or liveborn child with a neural tube defect. In the same period, there appeared to be a 40% higher probability of a neural tube defect in a female neonate or stillbirth than in a male one. Although, pre-fortification, the prevalence of neural tube defects was higher among indigenous neonates than among black, brown or white neonates, the differences in prevalence between black, brown and indigenous neonates were not statistically significant – probably because relatively few indigenous neonates were included in our analyses. Folic acid fortification appears to have lowered the prevalence of neural tube defects by 37.7% among the offspring of adolescent mothers and by 36.5% among female neonates and stillbirths.

**Table 3:** Neonatal and maternal characteristics and the prevalence of neural tube defects before and after mandatory folic acid fortification of flour, Brazil, 2001–2014

Characteristic	Pre-fortification^a^					Post-fortification^b^				Prevalence change %
	No. of births (% of total)	No. of NTD	Prevalence			No. of births (% of total)	No. of NTD	Prevalence		
			NTD per 1000 births	Ratio (95% CI)				NTD per 1000 births	Ratio (95% CI)	
**Maternal age, years ^c^**										
< 20	971 805 (19.5)	915	0.94	1.34 (1.2–1.4)		2 221 078 (17.0)	1 302	0.59	1.13 (1.1–1.2)	–37.7
20–34^d^	3 488 203 (70.0)	2 449	0.70	1		9 232 150 (70.5)	4 780	0.52	1	–26.3
> 34	526 589 (10.6)	371	0.70	1.00 (0.9–1.1)		1 640 707 (12.5)	938	0.57	1.10 (1.0–1.2)	–18.9
Total	4 986 597 (100.0)	–	–	–		13 093 935 (100.0)	–	–	–	–
**Sex of neonate or stillbirth^e^**										
Female	2 437 225 (48.8)	2 264	0.93	1.44 (1.3–1.5)		6 393 203 (48.8)	3 769	0.59	1.18 (1.1–1.2)	–36.5
Male^f^	2 560 657 (51.2)	1 656	0.65	1		6 718 656 (51.2)	3 368	0.50	1	–22.5
**Total**	4 997 882 (100.0)	–	–	–		13 111 859 (100.0)	–	–	–	–
**Race of neonate**^g^										
White	3 505 035 (77.4)	1 983	0.57	1.1 (1.0–1.2)		8 730 308 (69.4)	3 895	0.45	1.05 (1.0–1.1)	–21.1
Brown ^h^	880 425 (19.5)	439	0.50	1		3 347 507 (26.6)	1 422	0.42	1	–14.8
Black	122 622 (2.7)	73	0.60	1.2 (0.9–1.5)		454 023 (3.6)	204	0.45	1.06 (0.8–1.3)	–24.5
Indigenous	15 930 (0.3)	13	0.82	1.6 (0.9–2.8)		41 980 (0.3)	21	0.50	1.18 (0.8–1.8)	–38.7
**Total**	4 524 012 (100.0)	–	–	–		12 573 818 (100.0)	–	–	–	–

CI: confidence interval; NTD: neural tube defects.

^a^ The pre-fortification study period ran from 1 January 2001 to 31 December 2004 – although fortification became mandatory in June 2014.

^b^ The post-fortification study period ran from 1 January 2005 to 31 December 2014.

^c^ Maternal age was missing in 0.22% of the records.

^d^ Used as the reference category in the calculation of prevalence ratios for maternal age.

^e^ Sex of neonate or stillbirth was missing in 0.06% of the records.

^f^ Used as the reference category in the calculation of prevalence ratios for sex of neonate or stillbirth.

^g^ As no data on race were available for stillbirths, the data reported for race refer only to live births.

^h^ Used as the reference category in the calculation of prevalence ratios for race of neonate.

Although the pre-fortification and post-fortification study subjects were similar in terms of the sex distribution of the neonates or stillbirths, they differed slightly in terms of maternal age and neonatal race (Table 3).

## Discussion

Our finding of a decreased prevalence of neural tube defects after mandatory fortification of flour with folic acid confirms observations made in two previous Brazilian studies.^24^^,^^25^ One study investigated births in 19 hospitals that together represented 1% of all births in Brazil, and found a reduction in prevalence of 22.1% – from 1.04/1000 births pre-fortification to 0.81/1000 births post-fortification.^24^ The other study compared the prevalence of spina bifida in live births for one year pre-fortification and one year post-fortification, and reported a 39.1% reduction; 0.23/1000 to 0.14/1000 births.^25^ This study, however, included data from states where a substantial proportion of births are not recorded in the live birth database.^25^ Our study covers a longer period, a higher proportion of Brazilian births and all common forms of neural tube defects.

The reduction that we observed in the prevalence of neural tube defects in Brazil is higher than the corresponding reduction reported in the USA (19%),^17^ very similar to the reduction observed in South Africa (30.5%)^16^ and lower than the reductions reported in Chile (40%),^15^ Canada (46%)^14^ and – for anencephaly and spina bifida only – Argentina (58–60%).^24^ A meta-analysis of eight population-based studies on the fortification of flour or other foodstuffs with folic acid estimated the mean reduction in the prevalence of neural tube defects to be 46%.^11^ Although 78 countries have now made fortification of some foodstuffs with folic acid mandatory,^13^ only five have evaluated the effectiveness of such an intervention in the improvement of health outcomes.^11^

In our study, anencephaly was the most common type of neural tube defect, followed by spina bifida and then encephalocele. Although spina bifida has often been reported as the most common form of defect – followed by anencephaly and then encephalocele.^24^ Earlier reports on this topic have focused on live births whereas we investigated neural tube defects in both live births and stillbirths. It appears that, in Brazil at least, spina bifida occurs more frequently in stillbirths than in live births. The use of data from live births alone may lead to underestimates in the overall prevalence of neural tube defects.^19^^,^^20^

As seen in this and other studies,^14^^,^^26^^–^^28^ neural tube defects occur more often among female neonates and stillbirths than among males. Although the link between such defects and sex is probably complex, a female fetus requires more human chorionic gonadotropin than a male fetus and deficiencies in this hormone can increase the risk of neural tube defects. The neural tube closes in the first four weeks of embryonic development – i.e. well before the concentration of human chorionic gonadotropin in the fetus peaks on days 40–50 post-fertilization.^29^^,^^30^

In the present study, the prevalence of neural tube defects was found to be highest among indigenous neonates, closely followed by black neonates – although it should be noted that there were relatively few birth or death records of indigenous neonates. A study in Canada also found that the risk of neural tube defects was highest among indigenous neonates.^31^ Researchers in the USA found that the risk of neural tube defects in pregnancy was twice as high for women of Mexican descent than for white women.^32^

The effect of maternal age on the risk of neural tube defects is generally considered to be small. When any such association is observed, the prevalence of neural tube defects tends to be relatively high among those born to mothers in the youngest and oldest age groups.^33^ It is possible that the diets of adolescent mothers fail to satisfy the combined nutrient requirements for the growth of both the mother and her fetus. Another possibility is that, compared with older women, adolescent women are less likely to take supplements that contain folic acid.^34^ It remains unclear why older mothers might be at relatively high risk of giving birth to a child with a neural tube defect.

The public reporting of health data allows health planners to take evidence-informed policy decisions and helps foster a culture of accountability, transparency and efficiency.^35^^,^^36^ The relative completeness and reliability of Brazil’s live birth database and mortality information database were essential to the success of our study. Although the public reporting of demographic and health-care data in Brazil began in the 1970s, it was only after the implementation of the national public health system in the mid-1980s that the Brazilian Ministry of Health and other investigators concentrated efforts to guarantee the quality of the data being collected. There has been much evidence of the success of these efforts,^35^^,^^37^^–^^39^ including the results of a case–control study^40^ and active searches for unreported births and deaths.^41^^,^^42^ In 2010, the United Nations Children’s Fund acknowledged the political commitment to improving the health information systems in Brazil, especially those recording data on births and infant deaths.^43^ Brazil’s health information systems are now in line with the World Health Organization’s push towards evidence-informed policy.^44^

A possible source of bias in this study is the termination of pregnancy when the fetus is found to be anencephalic. Although abortion is illegal in Brazil, individual court rulings may override the law. However, there is so much bureaucracy involved in such cases that few abortions are permitted. We were unable to investigate – or control for – the effects of birth order, which is not recorded in either of the information systems we used. We assumed that children born in the second trimester of 2004 would not have benefited from maternal consumption of flour fortified with folic acid. It remains possible that some of these children did benefit but this would indicate that the benefits of fortification were even greater than those we presented.

## Conclusion

Our results show that the introduction of the mandatory fortification of flour with folic acid in Brazil was followed by a significant reduction in the incidence of neural tube defects in the centre, south-east and south of the country.
